# Meta-analysis of AKI to CKD transition in perioperative patients

**DOI:** 10.1186/s13741-021-00192-6

**Published:** 2021-06-29

**Authors:** Pedro M. Abdala, Elizabeth A. Swanson, Michael P. Hutchens

**Affiliations:** 1 Anesthesiology & Perioperative Medicine, Oregon Health & Science University, Portland, OR USA; 2 Medical Scientist Training Program, Oregon Health & Science University, Portland, OR USA; 3 Portland Veterans Affairs Medical Center, Operative Care Division, 3710 SW US Veterans Hospital Road, Portland, OR 97239 USA

**Keywords:** Surgery, Perioperative, acute kidney injury, Chronic kidney disease, Long-term outcomes of surgery

## Abstract

**Background:**

Recent research shows AKI increases the risk of incident CKD. We hypothesized that perioperative AKI may confer increased risk of subsequent CKD compared to nonperioperative AKI.

**Methods:**

A MEDLINE search was performed for “AKI, CKD, chronic renal insufficiency, surgery, and perioperative” and related terms yielded 5209 articles. One thousand sixty-five relevant studies were reviewed. One thousand six were excluded because they were review, animal, or pediatric studies. Fifty-nine studies underwent full manuscript review by two independent evaluators. Seventeen met all inclusion criteria and underwent analysis. Two-by-two tables were constructed from AKI +/− and CKD +/− data. The R package metafor was employed to determine odds ratio (OR), and a random-effects model was used to calculate weighted ORs. Leave-1-out, funnel analysis, and structured analysis were used to estimate effects of study heterogeneity and bias.

**Results:**

Nonperioperative studies included studies of oncology, percutaneous coronary intervention, and myocardial infarction patients. Perioperative studies comprised patients from cardiac surgery, vascular surgery, and burns. There was significant heterogeneity, but risk of bias was overall assessed as low. The OR for AKI versus non-AKI patients developing CKD in all studies was 4.31 (95% CI 3.01–6.17; *p* < 0.01). Nonperioperative subjects demonstrated OR 3.32 for developing CKD compared to non-AKI patients (95% CI 2.06–5.34; *p* < 0.01) while perioperative patients demonstrated OR 5.20 (95% CI 3.12–8.66; *p* < 0.01) for the same event.

**Conclusions:**

We conclude that studies conducted in perioperative and nonperioperative patient populations suggest similar risk of development of CKD after AKI.

**Supplementary Information:**

The online version contains supplementary material available at 10.1186/s13741-021-00192-6.

## Introduction

### Rationale

Clinical and translational studies suggest that incident acute kidney injury (AKI) leads to chronic kidney disease (CKD). A systematic review and meta-analysis by Coca et al. (Coca et al. 2012) demonstrated patients with AKI had higher risk for developing CKD with a pooled adjusted hazard ratio of 8.8 compared to patients without AKI. Development of CKD after AKI is heterogeneous in that it occurs in a variety of patient populations and can be caused by plethora of disease processes, including sepsis, cardiovascular disease, nephrotoxin exposure, and surgically induced stressors. Surgery can affect long-term outcomes of nonsurgical disease; for example, surgical patients may have elevated risk of cognitive dysfunction (Bratzke et al. 2018) occurring remotely from surgery itself. Perioperative patients experience AKI due to additional etiologies not present in nonsurgical patients (for example, exposure to iodinated contrast followed by cardiac surgery, renal artery occlusion in vascular surgery, nephrectomy in oncologic surgery, or desquamation and resuscitation in burn surgery). Thus, additional mechanisms impacting AKI-CKD transition may be present in perioperative patients. Therefore, surgical patients may be subject to differential renal injury and experience elevated risk of AKI-CKD transition. We hypothesized that perioperative status might confer differential risk of developing CKD after an AKI event. Since AKI-CKD transition occurs in a delayed fashion, it may be possible to intervene in the latent interval between resolution of AKI and clinical CKD. Given the burden of suffering imposed by CKD, risk stratification of patients with perioperative AKI is desirable and could ultimately improve care.

### Objectives

To perform subgroup analysis to determine whether risk of AKI-CKD transition varies according to perioperative or nonperioperative status.

## Methods

### Eligibility criteria

Studies published from 1975 to 2018 available in the English language were eligible for initial review. We excluded reviews, animal studies, and pediatric studies to select for studies involving adult human subjects.

### Information sources and search

MEDLINE search terms included AKI, CKD, chronic renal insufficiency, nephrotoxins, surgery, and perioperative.

### Study selection

We included studies in which:

1. Patients suffering AKI were included in the study
2. The study clearly defined AKI and CKD
3. The study excluded patients with prior CKD or separated patient data based on baseline kidney function (i.e., CKD stage, GFR) such that patients with CKD stage > 3 could be excluded from the analysis
4. The study allowed determination of perioperative status
5. The study stated CKD as an outcome
6. The study included data necessary for calculation of effect size

In studies in which patients with prior CKD were not excluded, but patient data was separated based on baseline kidney function, only patients with baseline (pre-AKI) GFR > 60 or stage 1–2 CKD were included in the data analysis. We defined incident CKD as CKD stage 3 or higher, according to the definition stated within each study.

### Data collection process and data items

Studies selected for final review were analyzed for the number of subjects in each of the categories:

1. Patients who suffered AKI and developed subsequent CKD
2. Patients who suffered AKI but did not develop CKD
3. Patients who did not suffer AKI but developed CKD
4. Patients who did not suffer AKI and did not develop CKD

Using these data, we calculated the OR for development of CKD in patients who suffered AKI vs. those who did not suffer AKI.

### Risk of bias in individual studies

We assessed risk of bias in individual studies using the Cochrane Collaboration’s tool for assessing risk of bias (Higgins et al. 2011). We did not assess performance bias or detection bias because there was no intervention applied to the patient populations being analyzed. Supplemental Table 1 presents the results of this analysis.

### Statistical analysis including summary measures, synthesis of results, risk of bias across studies, and additional analyses

Statistical analysis was conducted using R package metafor. The OR was calculated for each study using a random effects model to compute each weighted OR and subgroup ORs. Summary subgroup ORs were compared using a random effects model, meta-regression, and Wald analysis. Heterogeneity was assessed with the Cochrane *Q* test and *I*^2^. Leave-1-out and funnel plots were also used to assess the effect of heterogeneity and publication bias.

## Results

**Fig. 1:**
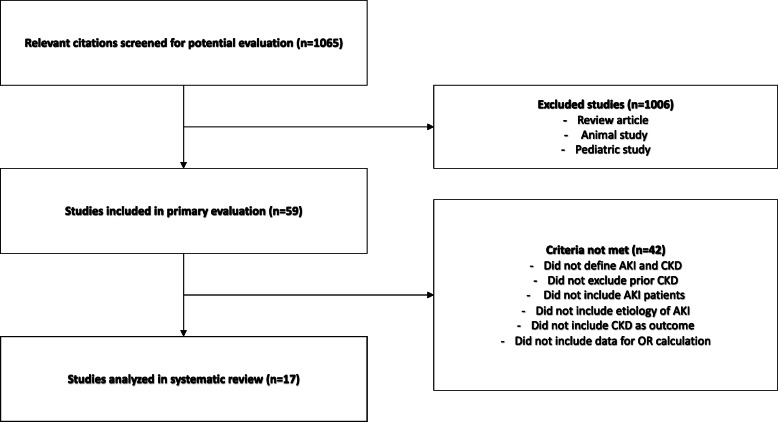
Explanation of studies included and excluded in final review

One thousand sixty-five studies were identified (Fig. 1). One thousand six studies were excluded after abstract review because they were animal studies, pediatric studies, or review articles. Fifty-nine studies underwent full manuscript review by two independent reviewers (PA, EAS). Seventeen studies fulfilled all inclusion criteria and underwent analysis. The Kappa measure of agreement between independent reviewers was 0.84 (*p* < 0.001). Disagreements about inclusion of studies in the meta-analysis were resolved by discussion with the senior author (MPH), resulting in exclusion of four studies from the meta-analysis. Individual risk of bias for the studies included was low for sixteen of seventeen studies and high in one study, as demonstrated in Supplemental Table 1.

Characteristics of the included studies are shown in Table 1. Ten of seventeen studies involved AKI in populations that were primarily perioperative (Wu et al. 2017; Palomba et al. 2017; Thalji et al. 2017; Legouis et al. 2017; Chew et al. 2017; Helgadottir et al. 2016; Arora et al. 2015; Xu et al. 2015; Rydén et al. 2014; Ishani et al. 2011). Of those, eight involved AKI in patients who underwent cardiac surgery (Wu et al. 2017; Palomba et al. 2017; Legouis et al. 2017; Chew et al. 2017; Helgadottir et al. 2016; Xu et al. 2015; Rydén et al. 2014; Ishani et al. 2011). The two remaining studies were in patients with burns (Thalji et al. 2017) and vascular surgery (Arora et al. 2015).

**Table 1:** Characteristics of the included studies

Study author	Sample Size	Definition of AKI	Definition of CKD	Population	Perioperative
Helgason et al. 2018	10885	KDIGO criteria	KDIGO 2012 CKD guidelines	Coronary angiography	No
Brown et al. 2016	24405	KDOQI guidelines	KDOQI guidelines	Cardiac catheterization	No
Chawla et al. 2011	11589	ICD9 codes	CKD stage 4 or higher	Pneumonia or MI	No
James et al. 2010b	920985	ICD9 codes	ESRD or doubling of serum Cr	Mixed medical etiologies	No
Ando et al. 2010	158	≥2x increase in serum Cr	KDOQI guidelines	Myeloablative allogeneic hematopoietic cell transplantation	No
James et al. 2010a	11249	Percent increase in serum creatinine	MDRD	Coronary angiography	No
Weiss et al. 2006	122	Percent decrease in GFR	Percent decrease in GFR > 25%	Non-myeloablative hematopoietic cell transplantation	No
Wu et al. 2017	1363	KDIGO criteria	KDIGO criteria	Cardiac surgery	Yes
Palomba et al. 2017	350	AKIN criteria	eGFR < 60 mL/min	Cardiac surgery	Yes
Thalji et al. 2017	18155	ICD9 codes	ICD9 codes	Burns	Yes
Legouis et al. 2017	4791	KDIGO criteria	eGFR < 60 mL/min	Cardiac surgery	Yes
Chew et al. 2017	3008	AKIN criteria	CKD stage 5	Cardiac surgery	Yes
Helgadottir et al. 2016	1754	KDIGO criteria	KDOQI guidelines	CABG	Yes
Arora et al. 2015	717	AKIN criteria	KDOQI guidelines	Endovascular or open surgical revascularization of lower extremities	Yes
Xu et al. 2015	3245	KDIGO criteria	KDIGO criteria	Cardiac surgery	Yes
Rydén et al. 2014	29330	AKIN criteria	Start of renal replacement therapy	CABG	Yes
Ishani et al. 2011	29330	AKIN criteria	Start of renal replacement therapy	CABG	Yes

Seven studies described AKI to CKD transition in nonperioperative populations. These included patients undergoing coronary angiography/cardiac catheterization (James et al. 2010a; Helgason et al. 2018; Brown et al. 2016), non-myeloablative hematopoietic cell transplantation (Weiss et al. 2006), myeloablative allogeneic hematopoietic cell transplantation (Ando et al. 2010), suffering from myocardial infarction or pneumonia (Chawla et al. 2011), or with mixed medical etiologies (James et al. 2010b).

### Quality assessment

Overall, there was significant heterogeneity across all studies (*Q* = 471.45, df = 16, *p* < 0.01; *I*^2^ = 98.3%). This was similar in perioperative studies (*Q* = 188.97, df = 9, *p* < 0.01; *I*^2^ = 93.7%) and nonperioperative studies (*Q* = 256.71, df = 6, *p* < 0.01; *I*^2^ = 97.9%).

### Effect size

**Fig. 2:**
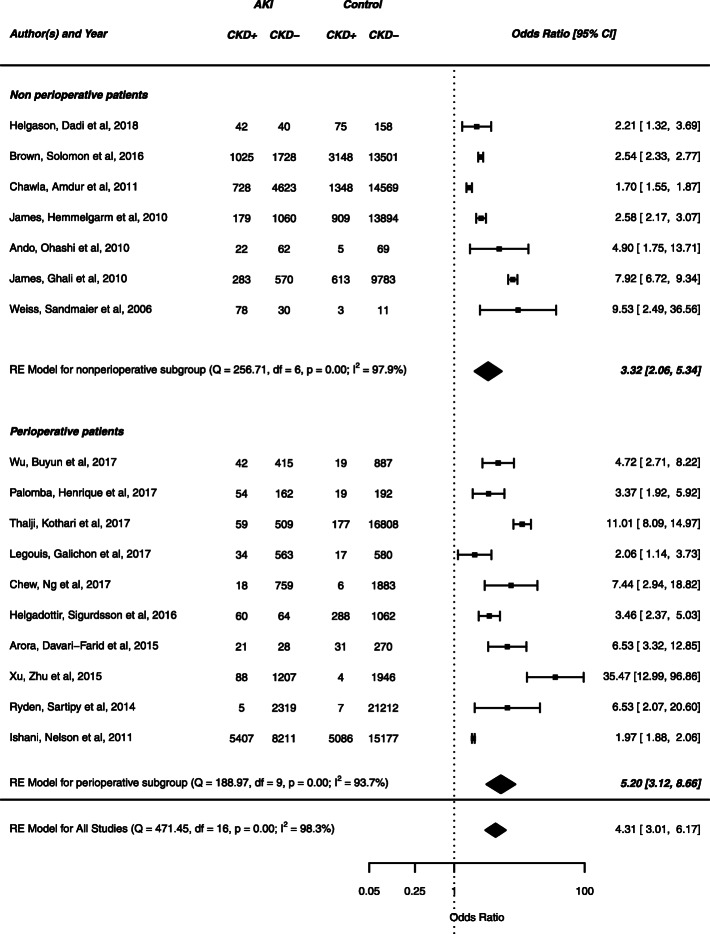
The effect size for each study included in the meta-analysis

Figure 2 depicts the effect size for each study included in the meta-analysis. Overall, AKI was associated with increased risk of subsequent CKD (OR = 4.31; 95% CI 3.01–6.17; *p* = 1.7 × 10^−15^). In the subgroup of studies of perioperative patients, the risk of new onset CKD was 5.2 times greater in patients with AKI than in those without (OR = 5.20; 95% CI 3.12–8.66; *p* = 2.9 × 10^−10^). In the subgroup of nonperioperative patients, the effect size was similar to that in perioperative patients (nonperioperative OR = 3.32; 95% CI 2.06–5.34; *p* = 8.0 × 10^−7^). The difference in effect size between perioperative and nonperioperative studies was not statistically significant. Therefore, in the studies reviewed, the risk of new onset CKD is elevated in patients who suffer AKI. Perioperative status confers at least the same risk as nonperioperative status.

## Discussion

The main finding of this meta-analysis is that studies conducted in perioperative populations demonstrate similar elevation of risk of AKI-CKD transition to those conducted in nonperioperative populations, with odds ratios of 5.20 and 3.32 respectively. Several groups have raised concerns that CKD could be a long-term outcome of perioperative AKI, and our data support this concern (Legouis et al. 2017; Palant et al. 2017). Numerous strategies to reduce the incidence of perioperative AKI are investigational (Romagnoli et al. 2018; Zarbock et al. 2018; Han and Lee 2019) and may therefore also potentially reduce postoperative development of CKD. Similarly, some have suggested patients with AKI receive surveillance for development of CKD (Fortrie et al. 2019); our results suggest this may be worthy of study in perioperative patients, in whom AKI may have a well-defined onset during a hospital stay, making them more amenable to intervention. It is widely speculated that intervention in the period between AKI and CKD development may ameliorate or even prevent CKD. For example, in cardiac surgery patients, with the advent of early biomarkers of AKI, it may be possible to predict incipient AKI as early as 6 h after surgery (Neyra et al. 2019), and 60% of AKI-CKD occurs within 1 year, while only 10% occurs within 160 days (Legouis et al. 2017). Therefore there is ample opportunity to identify at-risk patients and if possible, intervene. Although there is not yet a successful intervention, renin-angiotensin system inhibition, mediation of transforming growth-factor β and insulin-like growth factor, and antithrombin III have all been targets of investigation (Chou et al. 2018; Gao et al. 2020; Yin et al. 2017). Two clinical trials of interventions to prevent CKD after AKI (one of dietary intervention, NCT02831062 and one of intensified renal care, NCT4145609) are currently active. A critical translational step in monitoring patients after AKI would be development of specific biomarkers of AKI-CKD transition. Indeed, several have been proposed and recently reviewed (Wen and Parikh 2021); particularly hopeful may be urinary angiotensinogen, as this marker could also potentially be used to guide renin-angiotensin system based therapy (Cui et al. 2018). Thus, it is reasonable to speculate that with the right data, perioperative providers may be able to influence the course of AKI-CKD transition in the future. The present study suggests that perioperative patients have significant risk of AKI-CKD transition and points the way toward the data necessary for actionable knowledge.

A related secondary finding is that there is now a considerable dataset documenting AKI-CKD transition in many patient subgroups, such that subgroup analysis may be performed. We tested the hypothesis that perioperative status might modify risk, but other subgroup analyses are possible, and might further elucidate high risk populations. The present dataset does not allow determination of differential risk in specific surgical populations but suggests such studies are warranted and in the future could underpin risk stratification, potentially supporting intervention. For example, we note that two studies demonstrating lowest risk (Ishani et al. 2011, and Legouis et al. 2017), respectively evaluate CABG patients only and patients with low preoperative risk score (mean propensity-matched logistic EUROscore 4.3) and short cardiopulmonary bypass times (mean 79 min) consistent with a high proportion of CABG cases. Therefore, it is reasonable to speculate that patients requiring burn surgery and valve, heart transplant, or adult congenital heart surgery may have higher risk of AKI-CKD transition, while patients undergoing CABG alone may experience lower risk relative to that of more complex heart surgery. Further studies are warranted to address this potential signal. With additional studies of subpopulations of perioperative patients, risk stratification for surgical AKI-CKD may be possible.

Finally, we acknowledge this study has limitations. The primary limitation is significant heterogeneity across studies. To some degree, this should be expected given the broad nature of the subgroups included in this analysis. It should be noted that definitions of AKI varied among studies; this may be a source of heterogeneity. The nonperioperative subgroup in particular constitutes a broad collection of studies including various disease types and concurrently a broad patient population. A second limitation of the nonperioperative group is that it is not possible to ensure that all patients in nonperioperative populations did not have surgical exposure, increasing the risk of type 2 error. In perioperative studies, there was little diversity in the type of surgical procedures as nearly all studies of perioperative AKI-CKD have occurred in cardiac surgery patients who have high risk of AKI. This highlights an area for further investigation as risk seems likely to vary according to type of surgery. For example, same day/elective surgeries (e.g., cholecystectomy, appendectomy) may result in low-grade AKI (i.e., smaller changes in baseline creatinine) and thus may pose less risk of subsequent CKD compared to larger or emergent surgical procedures.

## Conclusions

We conclude that studies conducted in perioperative and nonperioperative patient populations suggest similar risk of development of CKD after AKI.

## Supplementary Information

**Additional file 1: Supplemental Table 1.** Results of assessment for bias.

## Data Availability

The datasets analyzed during the current study are available from the corresponding author on reasonable request.

## Abbreviations

AKI: Acute kidney injury
CKD: Chronic kidney disease
OR: Odds ratio
