# Supplementary material for: Meta-analysis of AKI to CKD transition in perioperative patients
Source: Perioper Med (Lond). 2021 Jun 29;10:24. doi: 10.1186/s13741-021-00192-6 (PMC8240318; doi:10.1186/s13741-021-00192-6)
Supplement: Supplementary file 1 — Additional file 1: Supplemental Table 1. Results of assessment for bias. [file 13741_2021_192_MOESM1_ESM.docx]

Supplemental Table 1

| **Study** | **Selection Bias** | **Attrition Bias** | **Reporting Bias** | **Overall Assessment** |
| --- | --- | --- | --- | --- |
| Helgason, Dadi et al, 2018 | Low | Low | High | Low |
| Brown, Solomon et al, 2016 | Low | Unclear | High | Low |
| Chawla, Amdur et al, 2011 | Low | Low | High | Low |
| James, Hemmelgarm et al, 2010 | High | Low | Low | Low |
| Ando, Ohashi et al, 2010 | Low | Low | Low | Low |
| James, Ghali et al, 2010 | Low | High | Low | Low |
| Weiss, Sandmaier et al, 2006 | Low | High | Low | Low |
| Wu, Buyun et al, 2017 | High | Low | Low | Low |
| Palomba, Henrique et al, 2017 | High | Low | Low | Low |
| Thalji, Kothari et al, 2017 | Low | Low | High | Low |
| Legouis, Galichon et al, 2017 | Low | Low | Low | Low |
| Chew, Ng et al, 2017 | Low | Low | Low | Low |
| Helgadottir, Sigurdsson et al, 2016 | High | Low | Low | Low |
| Arora, Davari−Farid et al, 2015 | High | Low | High | High |
| Xu, Zhu et al, 2015 | Low | Low | Low | Low |
| Ryden, Sartipy et al, 2014 | Low | Low | Low | Low |
| Ishani, Nelson et al, 2011 | Low | Low | High | Low |
